# The Toxicity of Poly(acrylonitrile-styrene–butadiene) Microplastics toward *Hyalella azteca* Is Associated with Biofragmentation and Oxidative Stress

**DOI:** 10.1021/acs.chemrestox.4c00300

**Published:** 2024-12-30

**Authors:** Lucas Gonçalves Queiroz, Caio César Achiles do Prado, Paulo Filho Marques de Oliveira, Daniel Farinha Valezi, Marcelo Cecconi Portes, Beatriz Rocha de Moraes, Rômulo Augusto Ando, Eduardo Vicente, Teresa Cristina Brazil de Paiva, Marcelo Pompêo, Bárbara Rani-Borges

**Affiliations:** † Institute of Biosciences, University of São Paulo, Rua do Matão 321, 05508-090 São Paulo, SP, Brazil; ‡ Engineering School of Lorena, University of São Paulo, Estrada Municipal do Campinho 100, 12602-810 Lorena, SP, Brazil; § Institute of Chemistry, University of São Paulo, Av. Prof. Lineu Prestes 748, 05508-900 São Paulo, SP, Brazil; ∥ Physics Department, State University of Londrina, Rodovia Celso Garcia Cid PR 445 Km 380, 86057-970 Londrina, PR, Brazil; ⊥ Department of Microbiology and Ecology, University of Valencia, Dr. Moliner 50, 46100 Burjassot, Spain

## Abstract

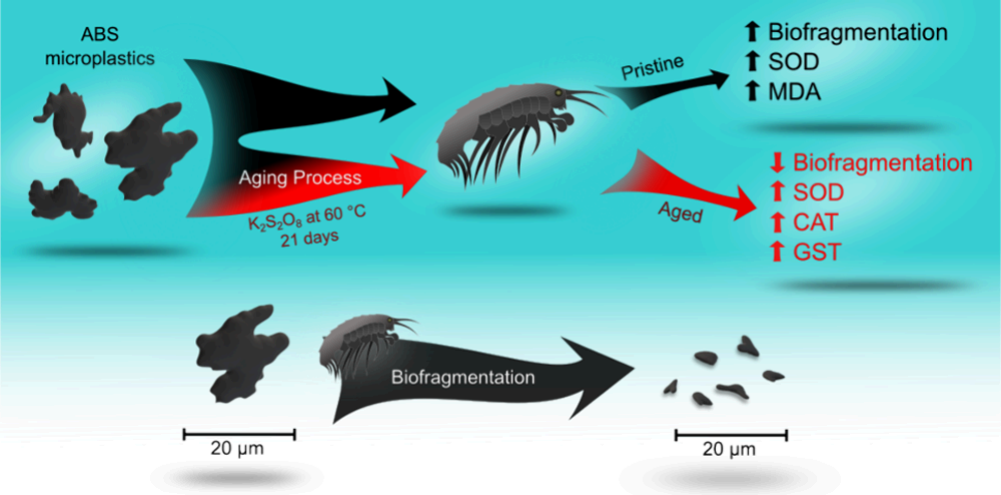

Acrylonitrile-butadiene-styrene (ABS) is a thermoplastic copolymer commonly used in the electronics, automotive, and construction industries. In the aquatic environment, the formation of microplastics from larger-sized plastic waste occurs naturally, induced by physical, chemical, and biological processes that promote the aging of these particles. Here, we investigated the interactions between the freshwater amphipod *Hyalella azteca* and ABS microplastics (10–20 μm) (pristine and after accelerated aging) over 7 days of exposure. At the end of the exposure period, we evaluated the ability of *H. azteca* to fragment the ABS particles, as well as the changes in its oxidative stress biomarkers (SOD, CAT, MDA, and GST) as the result of ABS exposure. *H. azteca* promoted a significant fragmentation of ABS particles. The ratio of this biofragmentation was more pronounced in pristine particles. Despite the absence of significant changes in the mortality of exposed organisms, alterations in the oxidative stress biomarkers were observed. The results demonstrate the ability of *H. azteca* to fragment pristine and aged ABS microplastics and, the consequent susceptibility of these organisms to the effects of microplastic exposure.

## 1 Introduction

Acrylonitrile-butadiene-styrene (ABS) is a thermoplastic copolymer widely used in the electronics, automotive, and construction industries.^1^ The ABS’ mechanical properties, dimensional stability, chemical resistance, synthesis, processing, and molding allow different applications of this copolymer.^2,3^ As a result of its widespread production and use, large amounts of waste materials containing ABS in their composition are improperly disposed of in the environment. For instance, ABS microplastics (<5 mm) have been detected in marine environments^4−6^ and freshwater bodies.^7,8^ Due to their small size, microplastic particles can be bioavailable in aquatic ecosystems and ingested by aquatic biota.^9,10^ Although there is evidence of ABS ingestion by aquatic organisms, the toxic effects caused by the exposure or possible interaction pathways between biota and ABS have not yet been investigated.

Another important aspect associated with the contamination of water bodies by microplastics is related to the aging of these polymeric particles.^11^ Polymer aging is related to the change of polymer properties over time as a result of physicochemical events, including temperature and mechanical stress as well as chemical reactions, such as unwanted cross-linking or radical generation and chain scission by the interaction with light.^12^ The aging process in the case of microplastics can be intensified by the large surface-to-volume ratio, i.e., a much higher surface area of the microparticles when compared to the bulk polymers. Therefore, once the microplastics reach the aquatic environment, they are subject to the effects of these natural aging agents (thermal and solar radiation, biodegradation, physical abrasion, etc.), which promote changes in their physical and chemical properties.^13^ These changes result in the aging of the polymeric particles and can accelerate the fragmentation process.^14^ Furthermore, it has been reported that for microplastics made of different polymers, the aging process can make them more hazardous to aquatic biota than the pristine material.^15,16^ Aged microplastics can induce several injuries on exposed organisms, such as increased lethality, oxidative stress, growth inhibition, behavior reduced, and impaired reproduction.

In the case of ABS, the available literature with information on the toxic effects promoted by ABS microplastics on the aquatic community is still scarce. In addition, studies that have sought to investigate the effects of aged ABS particles on aquatic organisms are nonexistent as far as we know. The insufficient data makes it difficult to comprehend the impact of this copolymer used worldwide on aquatic ecosystems. Additionally, the mechanisms of interaction between aquatic biota and plastic particles remain poorly documented, particularly the biofragmentation processes promoted by aquatic macroinvertebrates. Involuntarily, freshwater species ingest microplastics associated with the organic matter and digestive processes promote the fragmentation of these particles. The biofragmentation has already been reported by *Chironomus sancticaroli*, *Daphnia similis*, *Gammarus fossarum*, and *Hyalella azteca*.^17−20^

Seeking an integrated approach (action-response) in which we could obtain information about how macroinvertebrates and microplastics interact in the aquatic environment, the present study aimed to evaluate the toxic effects of ABS microparticles (10–20 μm), as well as the ability of the amphipod *Hyalella azteca* to promote the fragmentation of these particles. The organisms were exposed to pristine and aged ABS particles to understand how aging can affect their survival. We also could investigate the biofragmentation rates. Mortality and response of oxidative stress biomarkers were evaluated.

## 2 Experimental Procedures

### 2.1 Microplastic

**Figure 1:**
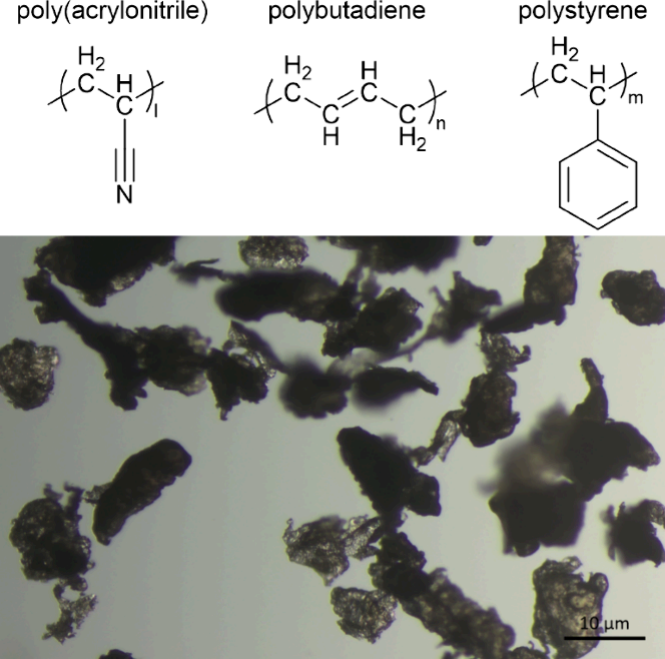
Chemical structure of ABS copolymer (top) and images from optical microscopy (bottom) of the irregular ABS particles (<20 μm) that were adopted in ecotoxicological assays using *Hyalella azteca*.

Acrylonitrile-butadiene-styrene (ABS) copolymer (Figure 1) was selected for the present study considering the limited research investigating its effects on freshwater macroinvertebrates. Although poorly studied, ABS is identified as one of the polymers with the greatest toxicological potential.^21^ For the present study, ABS filaments commercialized for 3D printing were cut into smaller pieces (∼0.5 cm) and washed with enzymatic detergent and 70% alcohol before cryo-milling. After the cryo-milling process, fragments were sieved to obtain particles between 10 and 20 μm. The known additives are carbon black pigment conferring the black color to the material (Figure 1) and calcium carbonate used during the production of ABS. Both substances are not expected to offer risks to aquatic organisms.

The size and shape of the ABS fragments were confirmed using a stereo microscope (Zeiss Discovery V12). A suspension containing ABS fragments was prepared in ultrapure water. The particle concentration of the suspension was confirmed by counting all the particles contained in 10 μL of the suspension. The average number of particles was 4933 ± 513 particles per mL. The counting was performed in triplicate under a stereomicroscope.

#### 2.1.1 ABS Microparticles Accelerated Aging

ABS was subjected to an artificial accelerated aging process using potassium persulfate (K_2_S_2_O_8_).^22^ Advanced oxidation processes (AOPs), such as treatments with persulfate, have the potential to degrade microplastics with high efficiency. When combined with other agents, such as UV irradiation, high temperatures, microwaves, and transition metals, they form the sulfate radical SO_4_^•–^, which can promote the oxidation of the polymer chains of plastic particles.^22−24^ ABS particles were treated in a glass bottle containing 30 mL of 1 M K_2_S_2_O_8_ at 60 °C for 21 days. The solution was renewed every 7 days. The ABS microplastic suspension was filtered in a fiberglass filter (47 mm diameter, 0.6 pore size) using a vacuum filtering system for the exchange solution. At the end of the treatment, the particles were washed several times with deionized water to remove any residue from the oxidizing agent.

#### 2.1.2 Physicochemical Characterization of Pristine and Aged Microplastics

The chemical composition of ABS particles was investigated by Fourier Transform Infrared Spectroscopy (FTIR). FTIR spectra were measured in a Bruker spectrometer, Alpha model, in the region of 400–4000 cm^–1^, with standard KBr beamsplitter and high sensitivity DLATGS detector. The spectra were recorded with the ATR (Attenuated Total Reflection) module: ATR Platinum, equipped with a germanium crystal as a reflective element.

The surface of pristine and aged microparticles was probed by X-ray Photoelectron Spectroscopy (XPS). XPS analyses were performed in Specs instrument in a fixed analyzer transmission mode, with excitation energy of Al Kα = 1486.71 eV, detector voltage of 1800 V, and Bias Voltage of 90 V. The spectra were acquired using a step size of 0.1 eV with a dwell time of 0.1 s, with an energy pass of 40 eV. Each spectrum is a result of 50 scans. The spectra were treated using CasaXPS software. C1s (aromatic/hydrocarbon) peak (284.9 eV) was used to calibrate the spectra for charge compensation. Peak fitting was carried out using a Shirley-type background and a combination of line shapes (GL).

Electron paramagnetic resonance spectroscopy (EPR) analyzed the formation of free radicals promoted by the aging process. EPR data were recorded in a CW-Bruker instrument, mod. EMX, operating at X-band (9.5 GHz, 20 mW power, 100 kHz frequency), using Wilmad quartz tubes, and DPPH (a,a′-diphenyl-*b*-picrylhydrazyl) as the frequency calibrant (*g* = 2.0036). Spectra were registered in the solid state at room temperature. The main parameters were: microwave power ≈2 mW, modulation frequency of 100 kHz, modulation amplitude of 5 G, and time constant of 20.48 ms. The same mass of pristine and aged ABS was used in the analyses.

EPR data were analyzed using Originlab 8.5 software. The *g*-factor was calculated using the resonance condition (*h*ν = *g*β*H*), while the comparison of the number of paramagnetic species was performed by calculating the area under the absorption signal (double integral of the resonance signal).

### 2.2 Exposure of *Hyalella azteca* to ABS Microplastics

In the present study, we used adult organisms of *H. azteca* (≈8 mm) from a continuous culture in the laboratory. The organisms were maintained at a controlled temperature (25 ± 1 °C) and fed three times a week with a suspension of Tetramin fish food (5 g·L^–1^). For the exposure tests, the organisms (*n* = 5) were placed in 600 mL glass bottles with metal caps and filled with 250 mL of MS culture medium, rich in mineral salts,^25^ and without aeration. Before starting the experiment, the organisms were subjected to an acclimation period without food and monitored for 48 h. The testing glass bottles containing the organisms were kept in the same photoperiod (16:8 h, light: dark) and temperature (25 ± 1 °C) conditions during the 7-day exposure period. Exposure of the organisms to ABS particles was done via food, following the method proposed by Rani-Borges et al. (2023). In a porcelain container, ABS microplastics at concentrations of 50 and 500 particles, called ABS1 and ABS2, respectively, were mixed with 100 μL of TetraMin suspension (5 g·L^–1^) and dried in an oven (60 °C for 24 h). Then, the containers were placed inside the testing bottles. Drying is an important step for this exposure route because it prevents the food and, consequently, the ABS particles from being rapidly released into the culture medium. For the negative control, porcelain containers contained only food. All treatments and the negative control were performed in triplicate.

### 2.3 Biofragmentation of ABS Particles

At the end of the exposure period of 7 days, the test solution containing ABS fragments was filtered through a glass fiber filter (47 mm, pore opening 0.5 μm) using a vacuum filtration system. The glass container was abundantly washed with filtered ultrapure water to remove any ABS particles that adhered to the vessel. The filter was drying at room conditions. Then, the filtrate was analyzed under a stereomicroscope (Zeiss Discovery V3) to count the number of particles formed at the end of the exposure period. For this, all particles present in an area of 285,000 μm^2^ of the filter were counted. Counting was performed in triplicate for each replicate. In the microscope analysis, the ABS particles were easily differentiated from the organic matter (remaining food or feces) in the sample due to the presence of carbon black pigment in their composition. As the food was offered only at the beginning of the exposure, we obtained clean samples at the end of the test. Thus, there was no need for further sample treatments such as the usual digestion of organic matter. Considering that particles may be fragmented due to their handling throughout the experiment, we performed a control particle treatment. Except for the presence of the organisms, control particles were subjected to identical experimental conditions. The biofragmentation ratio was determined from the following equation:
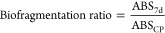


ABS_7d_ = average number of ABS particles obtained at the end of the 7-d experiment with *H. azteca*; ABS_CP_ = average number of ABS particles obtained at the control particles treatment.

### 2.4 Oxidative Stress Biomarkers

After 7 days of exposure, 3 organisms were used to prepare the homogenate (performed in triplicate). The organisms were transferred to 2 mL microtubes containing 600 μL of 100 mM potassium phosphate buffer, pH 7.4, and euthanized in a cold bath for 1 h. Using a glass rod, the organisms were macerated and then centrifugated at 4000 *g* for 30 min at 4 °C and stored under refrigeration (−80 °C). The protein concentration was determined according to the methodology proposed by Bradford (1976).^26^ The activity of SOD, CAT, and GST, as well as the levels of MDA, were determined as described by Queiroz et al. (2022).^27^

### 2.5 Mortality

The testing bottles were monitored on days 2, 4, and 7 to determine the survival rate of *H. azteca* organisms. At each observation, dead organisms were removed from the vessels. At the end of the exposure period (7 days), the percentage of mortality of the organisms was calculated compared to the control group.

### 2.6 Statistical Analysis

Data were expressed as mean ± standard deviation (SD). The statistical analyses were performed using MINITAB software. The results were submitted to Dunnett’s test and Tukey’s test to indicate significant differences between treatments and control groups (*p* < 0.05). The data obtained in the present study were subjected to principal component analysis (PCA) for data exploration and description. We adopted the Kaiser Rule which considers only the components that show eigenvalues greater than one (>1.0). Cluster analysis (CA) was performed seeking to find similarity patterns between treatments considering the Euclidian similarity index. PCA and CA were performed using Past 4.13 software.

### 2.7 Quality Control of Experiments

All the containers (porcelain containers, glass bottles, and vacuum filtering system) were rinsed at least three times with filtered ultrapure water before being used. Then, the containers were cleaned with cotton embedded with 90% acetone to remove microplastics. Also, all the containers were covered with aluminum foil to avoid contamination. The ultrapure water used for medium culture was filtered through 0.6 μm fiberglass prefilters (47 mm, 0.5 μm pore size) before use. To minimize environmental contamination, solution preparation, and sample manipulation were conducted in a fume hood. Blue nitrile gloves and white cotton lab coats were always adopted throughout the experiment as personal protection equipment. The different colors allow us to identify and differentiate any potential contaminant particles in the filters. After filtering, the filters were maintained in covered Petri glass dishes until stereomicroscope analysis.

## 3 Results and Discussion

### 3.1 Physicochemical Characterization of ABS Microparticles

**Figure 2:**
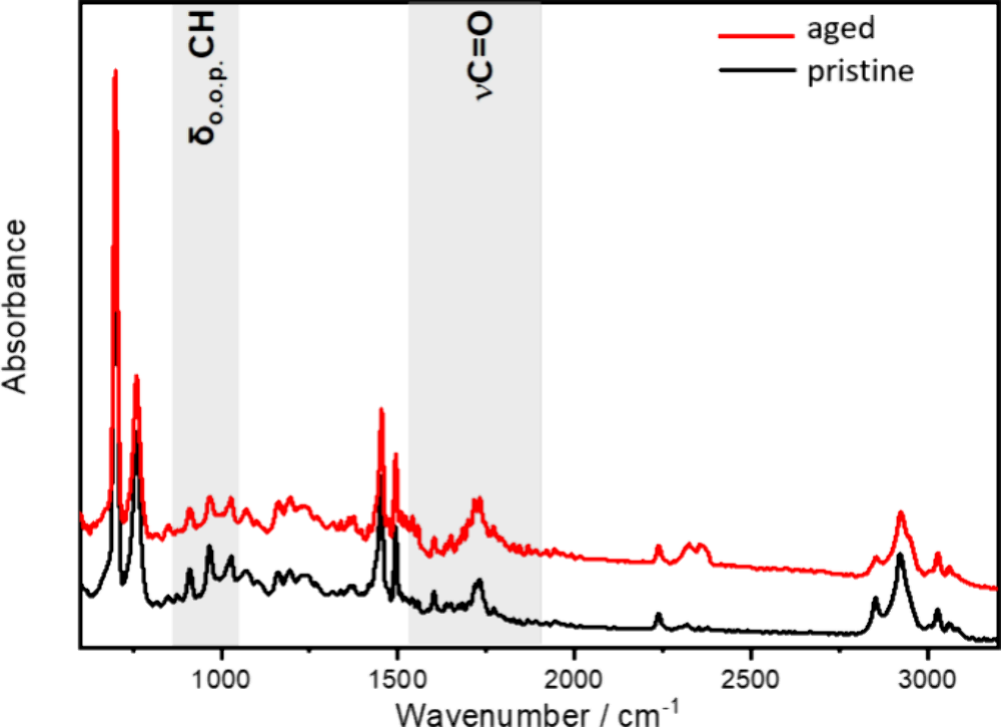
FTIR characterization of ABS particles in the region of 600–3200 cm^–1^ (ATR-Germanium).

The aging of the particles could be observed through the analysis of FTIR spectra. Vibrational analysis by infrared spectroscopy of polymers is an important tool to characterize structural alterations that occur during degradation and analyze the kinetics of the process by simulating natural weathering conditions. The microplastic aging method promoted the entry of oxygen into the ABS polymer structure, evidenced by the increased band at 1720 cm^–1^, as observed in Figure 2.

The oxidation of ABS is characterized by the formation of carbonyl groups with the simultaneous loss of unsaturation.^28^ By comparing the spectrum of pristine ABS with that of oxidized ABS through the infrared spectrum, it is possible to note the increased intensity of the band related to νC=O, 1720 cm^–1^. The oxygen present in the original pristine ABS is from the carbon black pigment. Thus, the aging can be evidenced by the increase of this band. Furthermore, it is possible to note an increase in the bandwidth (full width at half-maximum, fwhm) and the appearance of a shoulder at ca. 1680 cm^–1^ due to the formation of mixed products containing ketones, aldehydes, and esters groups. Concurrently, there is a decrease in the intensity of the bands attributed to the butadiene monomer unsaturations (δ_o.o.p._CH) at 910 and 965 cm^–1^, referring to the poly-1,2-butadiene and poly-1,4-butadiene units, respectively. A sharp decrease in intensity was observed for the 965 cm^–1^ band, showing that the oxidation process was effective in the main chain compared to the vinyl group present in the branching.^29,30^ Since butadiene provides toughness and ductility to the polymer, the process of breaking the unsaturated bonds can contribute to making the polymer more rigid.^31^

**Figure 3:**
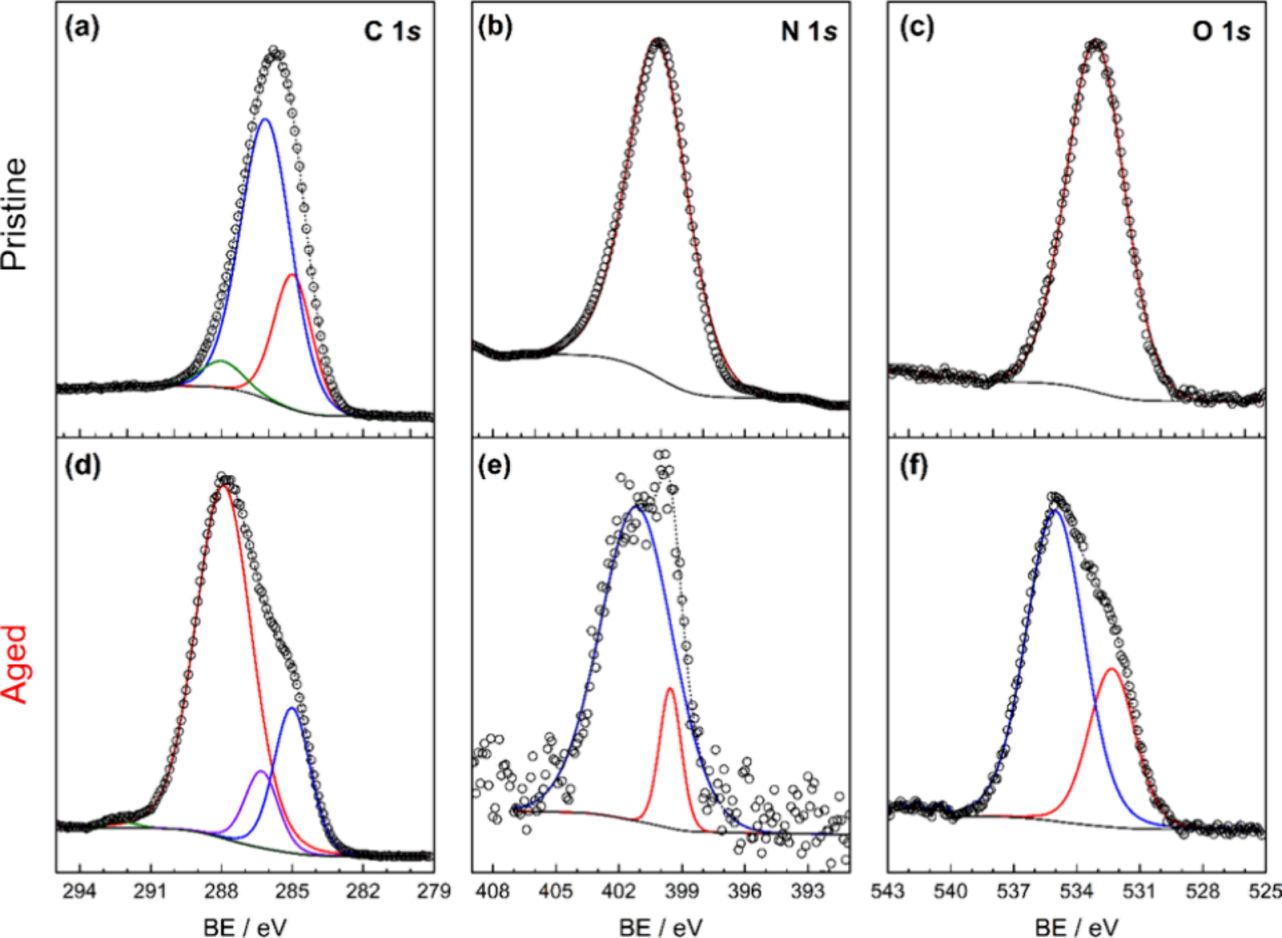
High-resolution XPS spectra of C 1s (a, d), N 1s (b, e), and O 1s (c, f) of pristine ABS (top-row) and ABS-aged (bottom-row).

X-ray Photoelectron Spectroscopy (XPS) is a surface-sensitive technique, which is appropriate to probe the chemical environment of materials through the binding energies (BE) related to specific core-level transitions. Figure 3 displays the XPS spectra from ABS before and after aging. For the pristine polymers (Figure 3a), peaks for C 1*s* can be seen at 284.9, 285.7, and 287.5 eV, which are attributed to C=C/C–C–H, C–O(H) and C=O/C≡N, respectively. The carbon–oxygen bonds in this case can be either from the already oxidized polymer itself and from the carbon black present in the sample as a pigment as well. The nontreated polymer also displays one single N 1s peak, from the C≡N (acrylonitrile moieties) at 399.7 eV (Figure 3b) and one O 1s at 532.8 eV (C–O bonds), possibly from the carbon black pigment, and at least in part as a result of the oxidation of the polymer backbone during the milling process. These values are in fair agreement with previous studies of XPS in polymers and carbon-based materials.^32,33^

When the sample undergoes an accelerated aging treatment, shifts in the binding energy and other peaks become apparent (Figure 3d–f). In the C 1s spectrum, peaks related to C=C/C–C–H and C–O are still present at 284.6 and 285.8 eV, respectively (Figure 3d). However, the peak at 287.6 eV becomes more intense, indicating an increase in carbon–oxygen species such as C=O. These results corroborate the data observed in the FTIR spectroscopy, as a result of the oxidative aging process. Additionally, a small peak at a higher binding energy (291.5 eV) appears, which can be attributed to carbon atoms in a more electronegative environment, meaning O–C=O, O–C(=O)O or even O–C(=N)O species.^34^ The results clearly indicate that the aging process produced polymers with a more oxidized (or aged) surface. In addition, for the ABS-aged N 1s spectrum (Figure 3e), there is a split in the peaks, giving BE of 399.4 eV, probably from the nitrile group, and another one at 400.7 eV. This higher energy BE can be attributed to C–N–(O)H bonds,^32,35^ which is also additional evidence of the aging process. Finally, the O 1*s* spectrum also displays two distinct peaks. The first at 532.2 eV, related to C–O bonds, and the second at 534.7 eV, generally related to H_2_O and O–C(=O)O species in certain configurations.^34^ Overall, the XPS results strongly demonstrate the effect of the aging process on the surface of the ABS microparticles.

**Figure 4:**
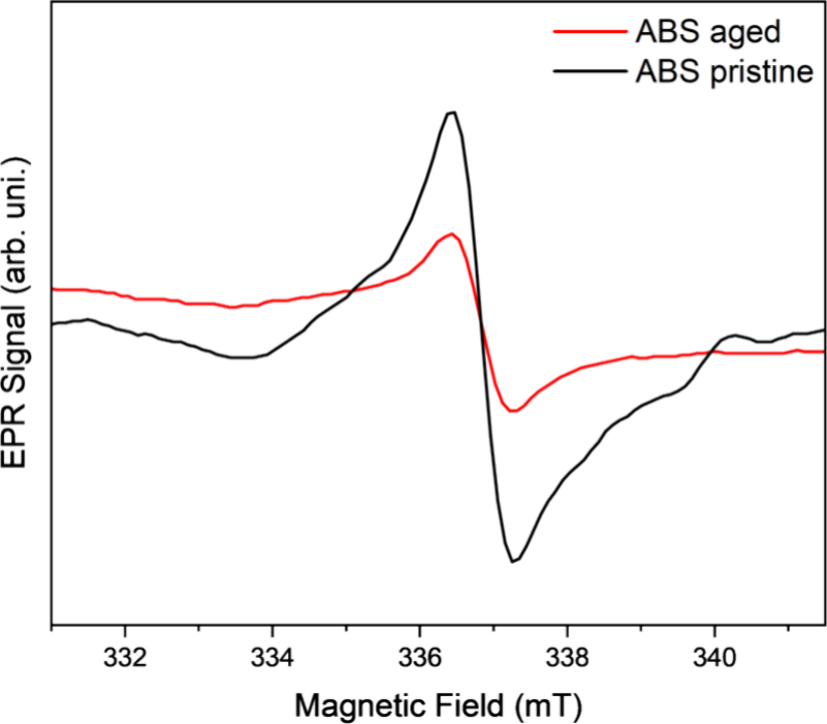
EPR spectra of ABS microplastics (pristine and aged).

One of the consequences of the aging process, either natural or induced, is the modification of the chemical nature of the particle surface, which is the first to be in contact with living organisms. This has already been demonstrated in the FTIR and XPS analyses. Additionally, we chose to further study the polymer microparticles using Electron Paramagnetic Resonance (EPR), targeting to probe the presence of radicals. Radical formation is very common in polymers which age predominantly through the propagation of radicals that catalyze chain scission. Radicals are typically induced by environmental UV irradiation. Figure 4 displays the EPR spectra of the pristine and aged ABS samples.

Both samples showed typical free radical EPR signals, with *g* = 2.001 and line-width Δ*H*pp = 0.88 mT. These parameters are in agreement with other free radical signals in ABS microplastic samples found in the literature.^36^ The striking difference between the spectra is in the signal intensity. The area under the integral of the EPR line curve is 2.1 times larger in the case of the ABS pristine. This indicates a significantly larger amount of free radicals present in such a sample (Figure 4). A similar increase was also observed in ABS microplastics in literature, in this case when exposed to light for 15 min.^36^

The presence of such radicals in ABS microplastic samples may be related to its conjugated benzene ring structure,^37^ and the *g* = 2.001 is typical of carbon-centered radicals.^38^

The smaller intensity in the EPR spectrum of aged microparticles (Figure 4, red curve) indicates that the aging process annihilated the original radicals of the pristine materials, resulting in a small signal related to them. When comparing this result with XPS (Figure 3), one can imply that the aging process contributed to the chemical modification of the polymer surface, by including additional oxygen and modifying the functional groups, and not by increasing the density of free radicals. A thermal treatment, with the same conditions but without potassium persulfate, was performed to assess the chemical action of potassium persulfate on the particles. This treatment reveals that the isolated thermal treatment promotes a higher formation of free radicals. Thus, the potassium persulfate seems to be reducing these radicals on the surface of the particles (Figure S1).

In the aquatic environment, microplastics are subjected to different natural agents, starting from the moment the particles are discharged. Thus, the aging of microplastics is an inevitable process, and consequently their degradation. However, the way aging occurs is dependent on a series of environmental factors and the polymeric characteristics of particles. Therefore, each particle, regardless of its composition, will undergo a unique aging process. In this study, we promoted the chemical aging of ABS particles using potassium persulfate. Among the aging indicators, the entry of oxygen into the polymeric structure of ABS was the most significant, as presented in Figures 2 and 3.

Santos et al. (2013) evaluated the influence of photo-oxidative degradation on ABS, under natural and simulated conditions. Similar oxidation rates, depending on the radiant exposure dosage, were found for ABS samples exposed to accelerated conditions and outdoors. Additionally, it was verified that the mechanical properties of ABS are affected by the formation of carbonyl groups and degradation of the butadiene component on the surface. The formation of cracks and their propagation within the copolymer also play an important role in the mechanical failure of ABS exposed to the tested conditions.^39^

Temperature and aging time are also important variables related to ABS aging. The thermal aging process results in intense oxidation in ABS particles.^40^ The copolymer aged by temperature can present changes in its mechanical properties, characterized by the formation of cracks due to the formation of carbonyl and hydroxyl on its surface.^41^ In the aquatic environment, on the other hand, photoaging seems to be the main responsible for promoting the oxidation of ABS particles and an increase in oxygen-containing functional groups. In addition, the long chains of the copolymer are broken down and released into the water.^42^ In this study, after exposure of ABS to potassium persulfate at 60 °C for 21 days, we also observed an increase in oxygen-containing functional groups in the polymeric structure of ABS (Figures 2 and 3), demonstrating that the proposed method was effective in promoting the aging of this copolymer.

It is important to highlight how ABS aging can vary significantly depending on the environmental factors to which the copolymer is exposed. Under natural conditions, where a range of variables act simultaneously and with varying intensities, the aging process can be intensified by combining these factors. Nevertheless, natural aging can take decades or hundreds of years to occur. Artificial aging, on the other hand, can accelerate this process and significant changes in the structure of microplastics can be observed in just a few days or months.^13^ Another point to be considered regarding the aging of microplastics is that aged particles have a greater tendency to adsorb other contaminants present in the environment.^11,43^ If we consider that aging is a natural process, one can imply that as the aging process advances, the toxicological potential of these particles is increased.

Adopting aged particles in ecotoxicological assays is particularly relevant from an environmental perspective. Once in the natural environment, microplastics are constantly exposed to biotic and abiotic factors that promote their aging.^13^ The interaction between microplastics and biota can result in toxic effects. On the other hand, the exposed organisms can act on these plastic particles promoting biofragmentation or biodegradation. In the present study, we adopted an action-response approach that could give information about the impacts of pristine and aged microplastics and how macroinvertebrates can interact with these plastic particles in the aquatic ecosystem.

### 3.2 Biofragmentation

**Figure 5:**
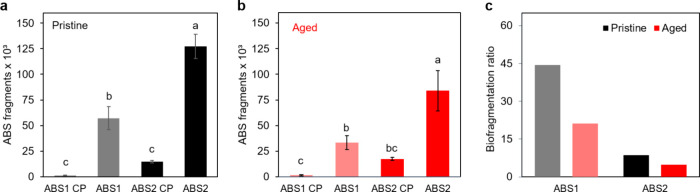
Fragmentation of ABS after 7-d exposure of *Hyalella azteca* to the pristine (a) and aged (b) ABS particles via food. Different letters indicate significant differences between groups (Tukey’s test, *p* < 0.05). Biofragmentation ratio (c) was obtained from average values obtained in the biofragmentation experiments.

The number of fragments observed at the end of the exposure period (7 days) was significantly higher in the treatments with *H. azteca* (ABS1 and ABS2) compared to the treatments without organisms (ABS1 CP and ABS2 CP) (Figure 5a and b) (*p* < 0.05). In addition, at the end of the exposure period, the ratio of biofragmentation of pristine ABS particles was higher than that of aged ABS. This increase was observed in both concentrations of ABS (ABS1 and ABS2) (Figure 5c). The results, therefore, demonstrate that *H. azteca* individuals were able to fragment ABS particles after 7 days of exposure.

Considering the quotient of the number of particles detected at the end of the test by the initial number, we observed that pristine ABS were 1.7 times more fragmented by *H. azteca* individuals than aged ones at the lowest concentration. At the highest concentration, the same pattern was observed with pristine microplastics being 1.8 times more fragmented (Figure S2).

Benthic invertebrates, such as the amphipod *H. azteca*, have great ecological relevance in aquatic environments. These organisms play a significant role in promoting organic matter cycling in these ecosystems.^44^ In general, amphipods have a mouth apparatus and a digestive system capable of promoting the grinding and fragmentation of particulate organic material, either by mechanical forces or by the action of enzymatic processes.^45,46^

The role of aquatic macroinvertebrates in the biofragmentation of microplastics in freshwater environments is not yet fully understood. Mateos-Cárdenas et al. (2020) reported the ability of *Gammarus duebeni* to promote, in a short exposure period (96 h), the biofragmentation of polyethylene spheres ranging in size from 10–45 μm, resulting in even smaller fragments until 0.5 μm. The ability of *H. azteca* to fragment microplastic has already been reported. In a previous study, we observed the ability of *H. azteca* individuals to ingest PS spheres (24.5 μm) and promote a reduction of these particles by 25% the diameter after 7 days of exposure to relevant environmental concentrations. Also, changes in the surface of the PS particles were observed.^18^ The present study on ABS microparticles follows the same trend and indicates microplastic particles were also ingested and fragmented by *H. azteca*. The microplastic particle size used in our tests ranged from 10 to 20 μm, and after the exposure period of 7 d, we observed a significant increase in the number of small particles (Figure S3). It is important to mention that there is an unavoidable formation of smaller particles due to handling along the sample preparation, including the control treatments. However, we observed that the number of ABS particles in the treatments with organisms was highly superior, indicating the role of *H. azteca* in the fragmentation of microplastics (Figure 5).

In general, we expected greater biofragmentation of the aged polymer, since the aging process can make the particles more fragile. However, the results obtained in the present study demonstrated a higher ratio of biofragmentation in treatments with pristine ABS (Figure 5c). This apparently controversial result can be explored by two hypotheses. The first hypothesis to explain the higher ratio of biofragmentation in the treatments with pristine ABS is related to the ability of *H. azteca* to identify and avoid exposure to harmful compounds or products generated by the aging process, i.e., a chemical aspect. According to Nguyen et al. (2012),^47^*H. azteca* individuals can recognize the presence of contaminants in their food and avoid it. According to Rummel et al. (2019),^48^ the aging of this copolymer can induce the formation of products from polymer chain scission that may have significant toxicological potential. Thus, the feeding selectivity of *H. azteca* is an important variable when adopting exposure via food, as in the present study. Therefore, the individuals used in this study may have avoided the food containing aged ABS particles offered at the beginning of the experiment. In Figure 5, it is possible to observe that the biofragmentation ratio was lower at higher concentrations (ABS2) in both treatments (pristine and aged ABS). In addition, aged ABS showed the lowest biofragmentation ratio at both concentrations tested. Thus, according to the hypothesis of feeding selectivity, we can suggest that aged ABS may present higher potential harm to these organisms, resulting from the artificial aging process.

Another important characteristic related to Hyalella feeding is associated with food texture. The second hypothesis for the lower biofragmentation ratio for aged ABS (Figure 5) is based on the physical aspect of the aged microparticles. The ingestion rate is directly affected by how easily *H. azteca* individuals can bite.^49^ The accelerated aging process of the polymer particles could have promoted cross-linking reactions. Free radical groups are formed during this process and new chemical bonds are generated, which was confirmed by the physicochemical characterizations (Figures 2 and 3). Thus, the hardness (and Young’s modulus) of plastic particles increases and the flexibility decreases, and so does ductility.^12,50^ In this context, it is also probable that the aged ABS polymer is more brittle than the pristine one. However, the digestive tract of *H. azteca* seems not to be able to break hard brittle items, considering that pristine and more flexible particles were fragmented at higher rates.

The reduction of feeding rates due to the feeding selectivity by Hyalella, induced either by chemical or physical factors, can negatively affect the development of these individuals and the population. Thus, our results suggest that in a microplastic-contaminated environment, even if there is no significant ingestion of particles, benthic macroinvertebrates may be negatively impacted due to food deprivation caused by selective feeding.

Finally, one more aspect concerning the fragmentation of the ABS particles can be related to the behavior of the *H. azteca* individuals in the benthic compartment. This organism is considered an important bioturbator of freshwater environments, including in the release of pollutants from the sediment.^51^ The ingestion and defecation by bioturbators, associated with mixing, burrowing, and reworking sediments, are considered important activities able to alter the physical properties of the benthic environments and ensure ecosystem services.^52^ Like any other particle of organic matter present in the sediment, microplastics are bioavailable for ingestion by aquatic organisms. Thus, the activities of these organisms can eventually promote changes in the structure of these particles. Particularly, flexible particles, such as pristine ABS, can be more susceptible to the effects of bioturbation. At the same time, it is expected that exposure to microplastics can induce biological responses. Thus, we included an integrated approach to obtain information about the action of an aquatic macroinvertebrate on ABS microplastics as well as the toxicological effects of microplastics on the organism.

### 3.3 Oxidative Stress

Exposure to xenobiotics triggers an immediate physiological response in exposed organisms. The metabolism of xenobiotics occurs by the action of enzymes in a process known as biotransformation which results in the formation of reactive oxygen species (ROS).^53^ Oxidative stress occurs when these ROS are not rapidly neutralized resulting in an imbalance between ROS and antioxidant enzymes. ROS can trigger damage to cellular structures, such as mitochondria, membrane lipids, DNA, structural proteins, and enzymes. Consequently, the organism becomes susceptible to opportunistic diseases.^54^

**Figure 6:**
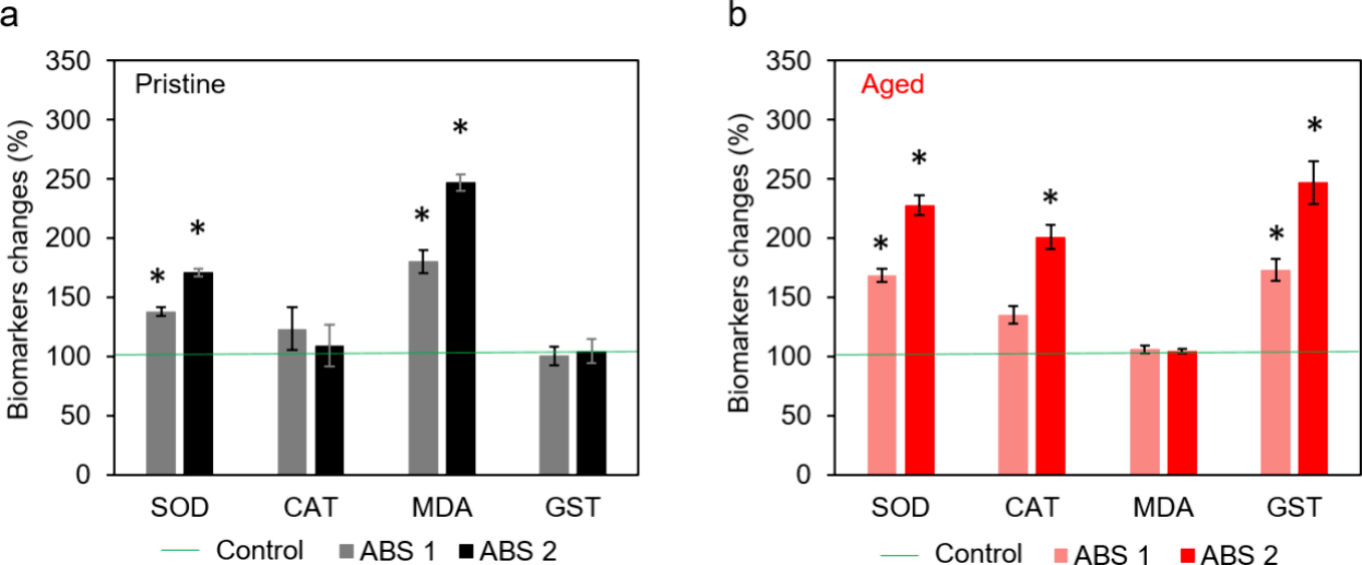
Oxidative stress (SOD, CAT, MDA, and GST) triggered after 7 d exposure to particles of ABS pristine (a) and aged (b). Asterisks (*) indicate a significant difference compared to the control group (100%) (Dunnett’s test, *p* < 0.05).

Exposure to ABS (pristine and aged) induced significant changes in the oxidative stress biomarkers evaluated in this study (CAT, SOD, GST, and MDA) (Figure 6). The pristine ABS promoted a significant increase in SOD activity and MDA levels compared to the control group. Moreover, these biomarkers were concentration-dependent in our tests, showing higher values at the highest concentration tested (Figure 6a). On the other hand, the aged ABS induced a significant increase in CAT, SOD, and GST activities (Dunnett’s test, *p* < 0.05) compared to the control group. The activity of these enzymes was also concentration-dependent (ABS2). The levels of MDA were not altered by aged ABS (Figure 6b).^55^

So far, no other studies have been found in the literature that evaluated the ability of ABS microplastics to promote oxidative stress in aquatic organisms. However, according to Farcas et al. (2019),^55^ ABS was shown to induce the formation of reactive oxygen species (ROS) in human cells. Their studies demonstrated cellular injuries and oxidative stress caused by ROS, consequently inducing the activity of antioxidant defenses, such as the total antioxidant capacity (TAC) and glutathione peroxidase (GPx). In the present study, both the pristine and aged ABS promoted a significant increase in SOD activity at both tested concentrations. Regarding CAT, only aged ABS caused changes at the higher tested concentration. SOD and CAT constitute the first line of defense in the xenobiotic detoxification process, controlling the formation of free radicals.^56^ The aged ABS also altered GST activity, which acts as the second line of defense by eliminating the products generated during detoxification.^57^ On the other hand, MDA is commonly associated with physical damage caused by microplastics on cells.^58^ Some studies have adopted MDA levels to evaluate oxidative stress by comparing pristine and aged microplastics. They have observed an increase in this biomarker in both conditions (pristine and aged).^59,60^ In the present study, we only observed increased MDA levels in the pristine ABS treatment (*p* < 0.05).

Considering that the aging process promotes fragility in the microplastic structure,^11^ the pristine particles can be more flexible and, consequently, more susceptible to biofragmentation, causing more physical damage to the digestive tract of the organisms when ingested. Smaller plastic microparticles were produced by *H. azteca* in the presence of pristine ABS (Figure 5c), indicating that this type of microplastic was also more consumed, and therefore, physically damaging the organism, and consequently increasing MDA levels. In other words, the increased level of MDA for the tests performed with pristine ABS can also have a significant contribution from the biofragmentation process by the respective organisms.

Although our hypothesis considers that *H. azteca* can avoid contaminants by feeding selectivity, we believe that some of the microplastics could be ingested because ABS particles and food were offered together. In this case, the inescapable ingestion of microplastics, however without extended fragmentation by the organisms, is the most plausible explanation for changes in oxidative stress biomarkers (SOD, CAT, and GST) and the low biofragmentation rates in the aged ABS treatment (Figure 6).

An important characteristic of AOPs on plastic polymers is the production of free radicals.^61^ The oxidative stress biomarkers are relevant indicators of the effects of these radicals on living organisms. We suggest that this information can corroborate the increase in biomarker levels, mainly after the aging process (Figure 6). The action of these enzymes is important to avoid significant physiological damage promoted by hydroxyl (^•^OH) and superoxide (O_2_^•^) radicals. These radicals are very highly harmful and reactive.^62^

Considering the EPR results (Figure 4), the observed oxidative stress can not be attributed to the possible free radicals formed during the aging process, because the pristine ABS showed a higher concentration of radicals than the aged ones. The formation of radicals resulting in the observed oxidative stress can be attributed to endogenous detoxification mechanisms. Thus, considering that the aged microparticles have a lower concentration of free radicals at the beginning of the tests (as shown by EPR) when compared to pristine particles, the increased biomarker levels for the aged ones can be attributed to the changes in the functional groups on the polymer surface, as demonstrated by XPS. These more oxidized groups are also sites for oxygen delivery, and therefore, a possible site for the generation of radicals or toxic chemical species such as C≡N to C–N–OH (oximes) during the tests.

### 3.4 Survival

Exposure to ABS particles (pristine and aged) for 7 days did not significantly affect the survival of *H. azteca* (Dunnett’s test, *p* < 0.05) (Table S1). Thus, the aging of ABS, under the conditions adopted in this study, did not prove to be a determining factor capable of affecting the survival of *H. azteca*. According to the literature, ABS toxicity does not seem to be associated with a reduction in the survival of aquatic organisms exposed to it. For example, *Daphnia magna* individuals exposed to ABS did not have their survival rates reduced, even at the highest tested concentration of 260 g·L^–1^.^21^ The survival of *H. azteca* has also been reported for other polymers, leading to similar results. For instance, PET particles (32–38 μm) did not cause mortality in *H. azteca* at environmentally relevant concentrations, although they did promote oxidative stress in exposed organisms.^27^ In another study, PS particles (20–500 μm) at concentrations up to 40% in dry sediment did not cause significant effects on *H. azteca* individuals.^63^ On the other hand, high concentrations of PE (10–27 μm), above 10,000 particles·mL^–1^ resulted in the mortality of *H. azteca*.^64^ The absence of mortality in this study can be attributed to the low concentrations tested, which approximate the realistic concentrations of microplastics found in aquatic environments,^65^ thus, representing an environmentally relevant approach. Higher concentrations could be tested to induce mortality, although those would not represent what is observed in the natural environment.

### 3.5 Data Analysis

**Figure 7:**
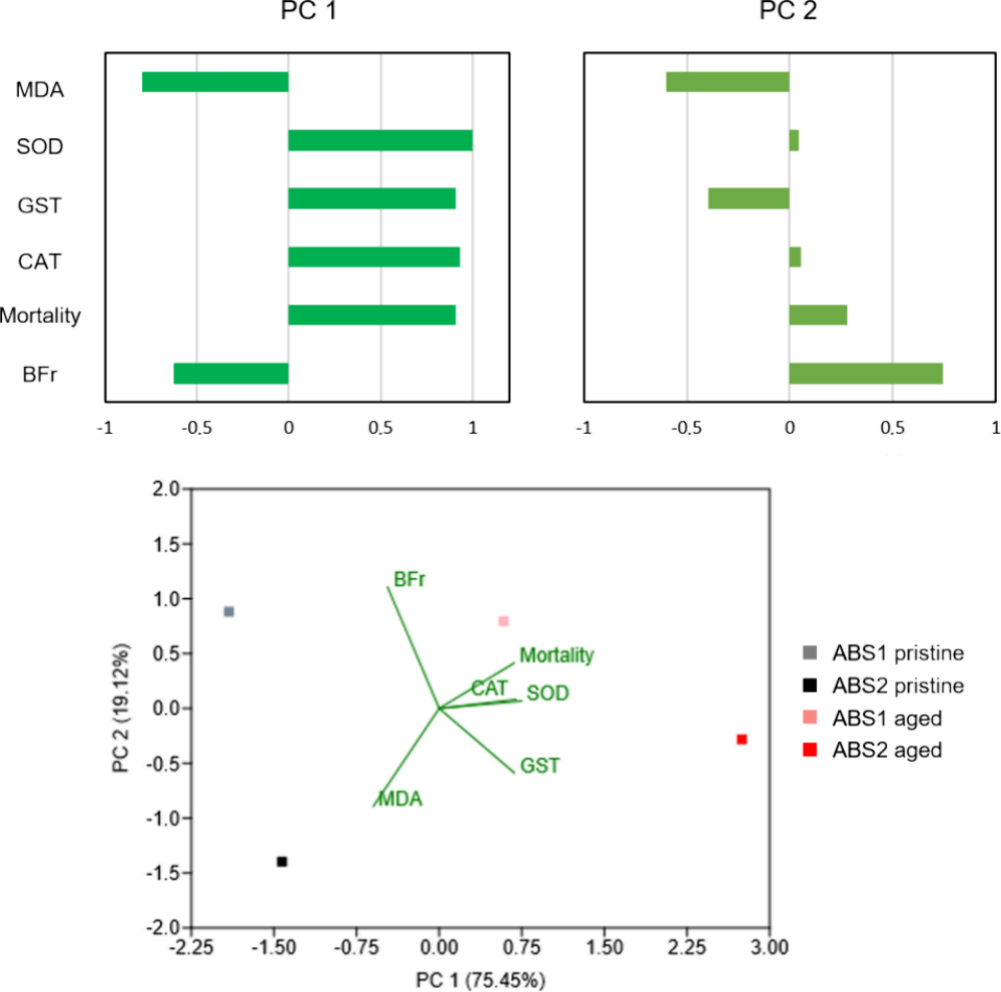
Principal component analysis (PCA) score and loading plots of the variables obtained from the exposure to ABS (pristine and aged) particles.

PCA was carried out for the data on biofragmentation rate, mortality, and oxidative stress biomarkers. The results can assist in understanding the main variables related to the effects of ABS (pristine and aged) particles on *H. azteca*. As a result, two components (PC1 and PC2) were extracted, with component 1 (PC1) explaining 75.45% of the total variance and component 2 (PC2) contributing an additional 19.12%. Both components displayed a cumulative contribution rate of 94.57%. The distribution of the loads for each variable can be observed in Figure 7.

The variables that showed large loads on PC1 (Mortality, CAT, GST, and SOD) were related to ABS2 aged. These results reinforce the potential of aged ABS particles to affect *H. azteca*. Biofragmentation (BFr) showed a larger load on PC2, 0.694. Particularly, high levels of biofragmentation were observed at low ABS concentrations in both treatments (pristine and aged) (Figure 5). PC1 also evidenced the relation between biofragmentation and MDA levels. Both variables were increased in the treatments using pristine ABS microplastics which reinforces the hypothesis that the increase in MDA levels could be a response triggered by a facilitated and increased consumption of flexible particles, yet able to promote physical damage.

Cluster analysis of the treatments (pristine and aged) represents the similarities and dissimilarities considering the variables determined in the present study. Corroborating PCA, CA also evidenced the differences between pristine and aged treatments. Additionally, we observed the similarities between low and high concentrations of the same treatment. The dendrogram showing formed clusters is displayed in Figure S4.

## 4 Conclusions

The effect of ABS polymer on *H. azteca* was studied, probing both the mortality of the individuals and the bioindicators of the potential toxicity of the microplastics on this living organism. Aiming to mimic natural processes, the ABS microparticles (10–20 μm) were aged under accelerated conditions using persulfate. Thermal stress associated with potassium persulfate contributed to the aging process, which was confirmed by FTIR and XPS analysis. *H. azteca* individuals promoted an expressive fragmentation of ABS microplastics within 7 days. Fragmentation was more pronounced in the pristine ABS, compared to the aged ABS treatment. Aspects related to the chemical changes of the particles’ surface generated by aging, as well as the ability of the organisms to recognize and avoid potential toxic agents in their food as well as the hardness of aged particles, explain the difference in biofragmentation rates. Although no significant mortality was observed, the exposure to both pristine and aged ABS triggered oxidative stress, which was confirmed by changes in the activity of detoxification biomarkers (SOD, CAT, MDA, and GST). The physical damage indicated by MDA levels seems to be related to the higher fragmentation ratio of the pristine ABS microparticles.

Our results indicate that the physicochemical properties of the microparticles can play a crucial role in the benthic environments, herein illustrated by the response of aquatic macroinvertebrates. The study also demonstrated the importance of polymer aging processes, which naturally occur in the environment. A comprehensive investigation into the mechanisms of aging and cross-linking reactions within polymer microparticles is essential to enhance our understanding of the associated chemical changes and related impacts in natural environments. The combination of physicochemical characterization of microplastics and ecotoxicology is indispensable for achieving a more complete picture of microplastics’ pollution. Such an approach would enable a more thorough assessment of the changes in the mechanical and chemical properties of aged microplastics, with a particular emphasis on strength, toughness, internal structure, and surface structure, to comprehend their impact on brittleness and the related environmental risks.

## Supporting Information Available

Additional information about survival, EPR analysis, biofragmentation microscopy, and cluster analysis (PDF)
