# Supplementary material for: The Toxicity of Poly(acrylonitrile-styrene–butadiene) Microplastics toward Hyalella azteca Is Associated with Biofragmentation and Oxidative Stress
Source: Chem Res Toxicol. 2024 Dec 30;38(1):91–101. doi: 10.1021/acs.chemrestox.4c00300 (PMC11752492; doi:10.1021/acs.chemrestox.4c00300)
Supplement: Supplementary file 1 — tx4c00300_si_001.pdf [file tx4c00300_si_001.pdf]

Supporting Information

The toxicity of poly(acrylonitrile-styrene-butadiene) microplastics towards *Hyalella azteca* is associated with biofragmentation and oxidative stress

Lucas Gonçalves Queiroz [1*], Caio César Achiles do Prado [2], Paulo Filho Marques de Oliveira [3], Daniel Farinha Valezi [4], Marcelo Cecconi Portes [3] Beatriz Rocha de Moraes [3], Rômulo Augusto Ando [3], Eduardo Vicente [5], Teresa Cristina Brazil de Paiva [2], Marcelo Pompêo [1], Bárbara Rani-Borges [3**]

[1] Institute of Biosciences, University of São Paulo, Rua do Matão 321, 05508-090, São Paulo, SP, Brazil.

[2] Engineering School of Lorena, University of São Paulo, Estrada Municipal do Campinho 100, 12602-810, Lorena, SP, Brazil;

[3] Institute of Chemistry, University of São Paulo, Av. Prof. Lineu Prestes 748, 05508-900, São Paulo, SP, Brazil;

[4] Physics Department, State University of Londrina, Rodovia Celso Garcia Cid PR 445 Km 380, 86057-970, Londrina, PR, Brazil;

[5] University of Valencia, Department of Microbiology and Ecology, Dr. Moliner 50, 46100, Burjassot, Spain.

Corresponding authors:

* Lucas Gonçalves Queiroz, University of São Paulo, Institute of Biosciences, Rua do Matão, 321, São Paulo, SP, Brazil. (queiroz@ib.usp.br)

** Bárbara Rani-Borges, University of São Paulo, Institute of Chemistry, Av. Prof. Lineu Prestes, 748, São Paulo, SP, Brazil. (barbara.rani-borges@usp.br)

Items

Figure S1. EPR spectra of samples ABS (pristine, aged, and after thermal treatment).. S3

Figure S2. The quotient of the number of ABS particles detected at the end of the test by the initial concentration……………………………………………………………S4

Figure S3. Small ABS microplastics fragmented by *Hyalella azteca* after 7 d exposure……………………………………………………………………………...S5

Figure S4. Dendrogram showing clusters in treatments considering the endpoints analyzed in the present study (Biofragmentation (BFr), mortality, MDA levels, GST, SOD, and CAT activities)……………………………………………………………....S6

Table S1. Mortality (%) of *Hyalella azteca* exposed to ABS microplastics (pristine and aged) after 2, 4, and 7 days of exposure………………………………………………S7

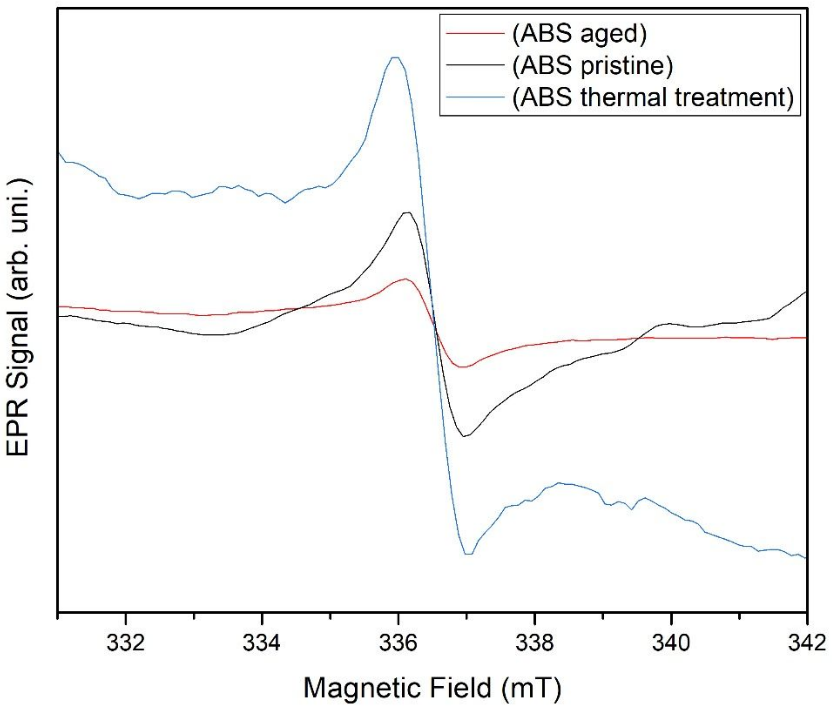

Figure S1: EPR spectra of samples ABS (pristine, aged, and after thermal treatment).

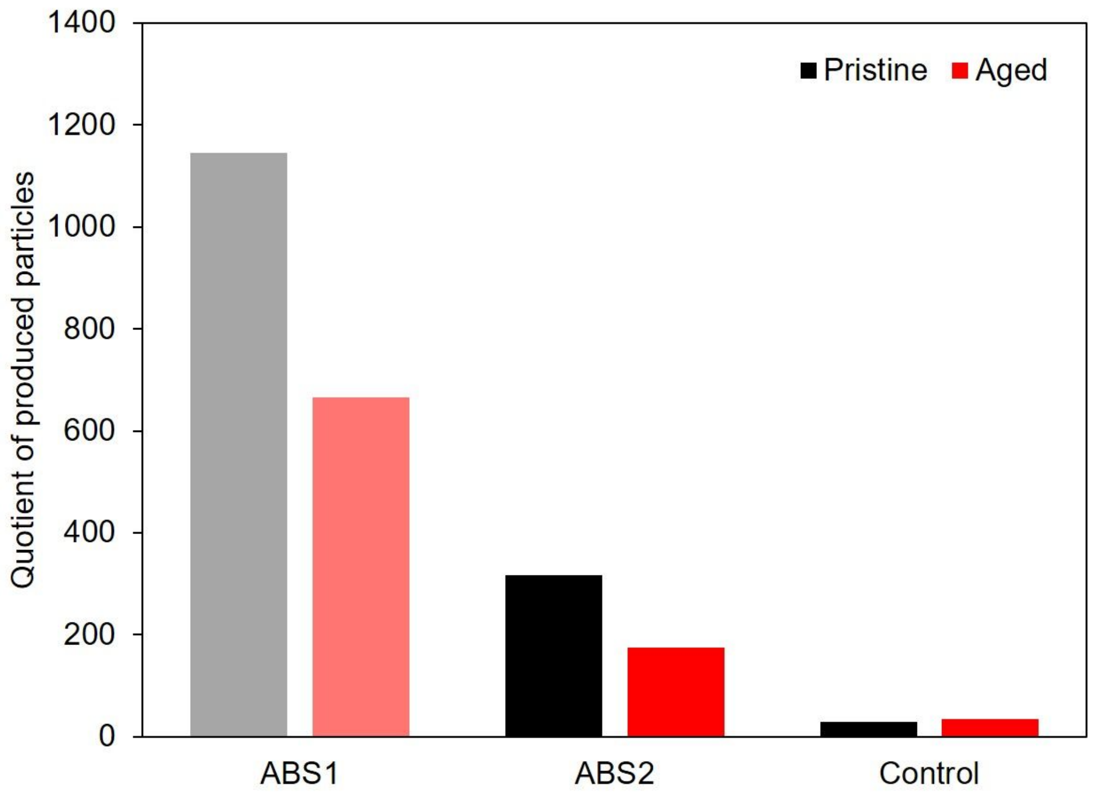

Figure S2. The quotient of the number of ABS particles detected at the end of the test by the initial concentration. Particle control was performed with the same concentration of the ABS2 treatment (500 particles).

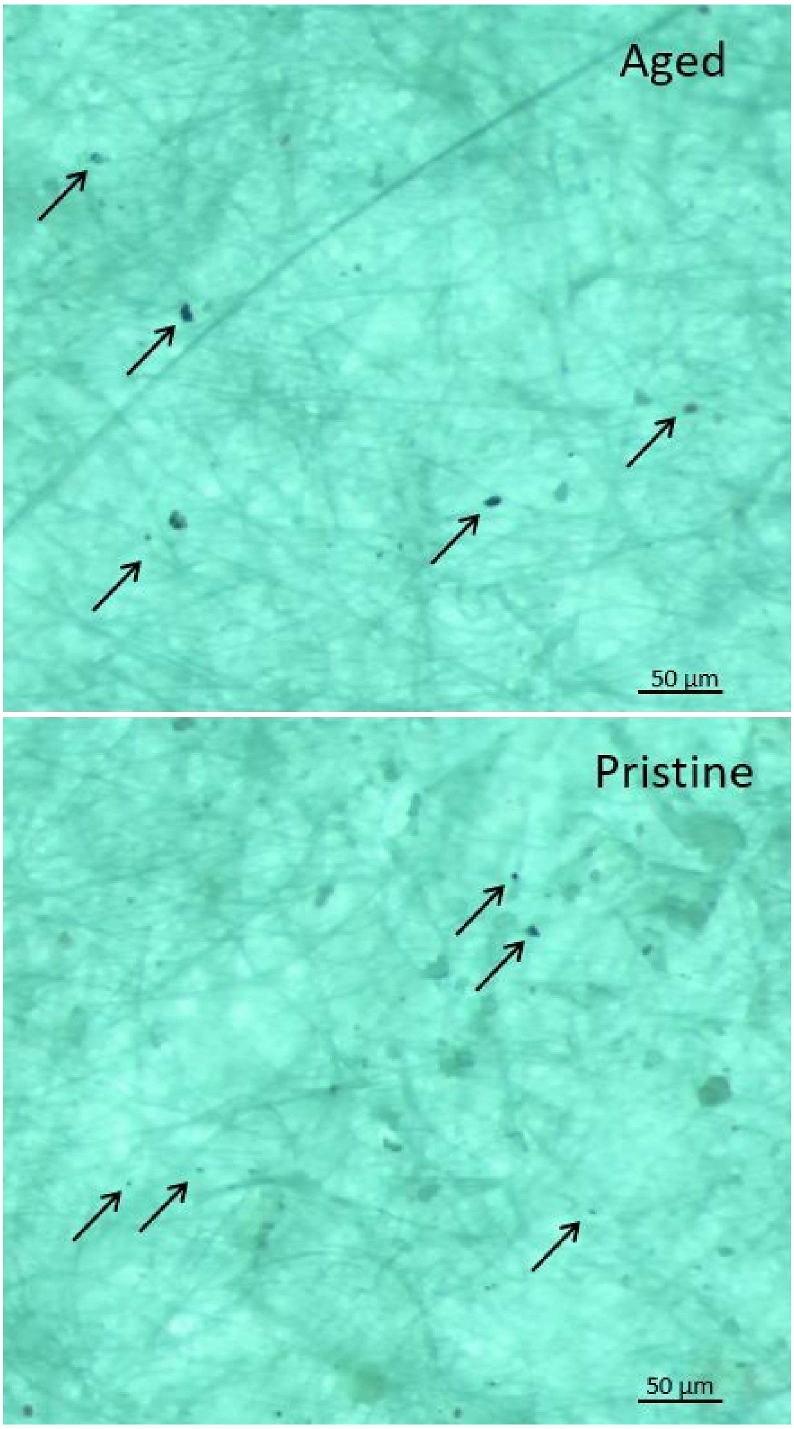

Figure S3. Small ABS microplastics fragmented by *Hyalella azteca* after 7 d exposure.

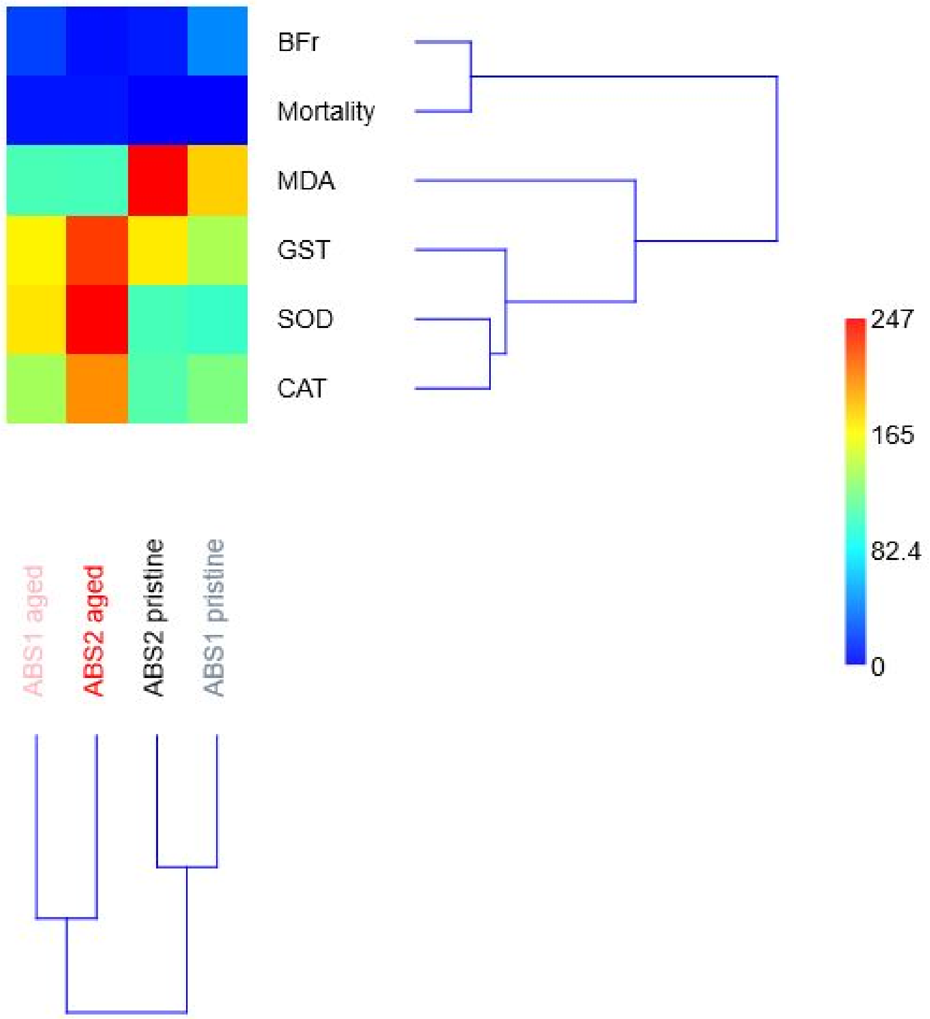

Figure S4. Dendrogram showing clusters in treatments considering the endpoints analyzed in the present study (Biofragmentation (BFr), mortality, MDA levels, GST, SOD, and CAT activities).

Table S1. Mortality (%) of *Hyalella azteca* exposed to ABS microplastics (pristine and aged) after 2, 4, and 7 days of exposure.

	**ABS pristine**			**ABS aged**		
**Treatment**	**2d**	**4d**	**7d**	**2d**	**4d**	**7d**
**Control**	0.0	0.0	0.0	0.0	0.0	6.67 ± 0.0
**ABS1**	0.0	0.0	0.0	0.0	6.67 ± 0.0	6.67 ± 0.0
**ABS2**	0.0	0.0	0.0	6.67 ± 0.0	6.67 ± 0.0	6.67 ± 0.0
